# Dr. Gustav Jaeger

**Published:** 1917-07

**Authors:** 


### OBITUARY

**Readers are requested to report promptly the death of all physicians in Western New York, or former residents of this region, or graduates of any medical school in Western New York, and to notify the families of the deceased of our desire to publish adequate Obituary notices.**

Of 116 death notices published in the last year, 15 were of physicians or scientists of renown, or of persons not themselves physicians but well known to the local profession. 101 were of physicians who either resided in our territory or had previously practiced in it or had graduated from Western N. Y. medical schools.

**Dr. Gustav Jaeger,** the inventor of special woolen underwear, died in May in Stuttgart, having been born in Wurtemberg in 1833.

